# Supplementary material for: Transport and inhibition mechanism of the human SGLT2–MAP17 glucose transporter
Source: Nat Struct Mol Biol. 2023 Dec 6;31(1):159–69. doi: 10.1038/s41594-023-01134-0 (PMC10803289; doi:10.1038/s41594-023-01134-0)
Supplement: Supplementary file 1 — Supplementary Figs. 1–10 and Tables 1 and 2. [file 41594_2023_1134_MOESM1_ESM.pdf]

nature structural & molecular biology

Article https://doi.org/10.1038/s41594-023-01134-0

Transport and inhibition mechanism of the human SGLT2–MAP17 glucose transporter

In the format provided by the authors and unedited

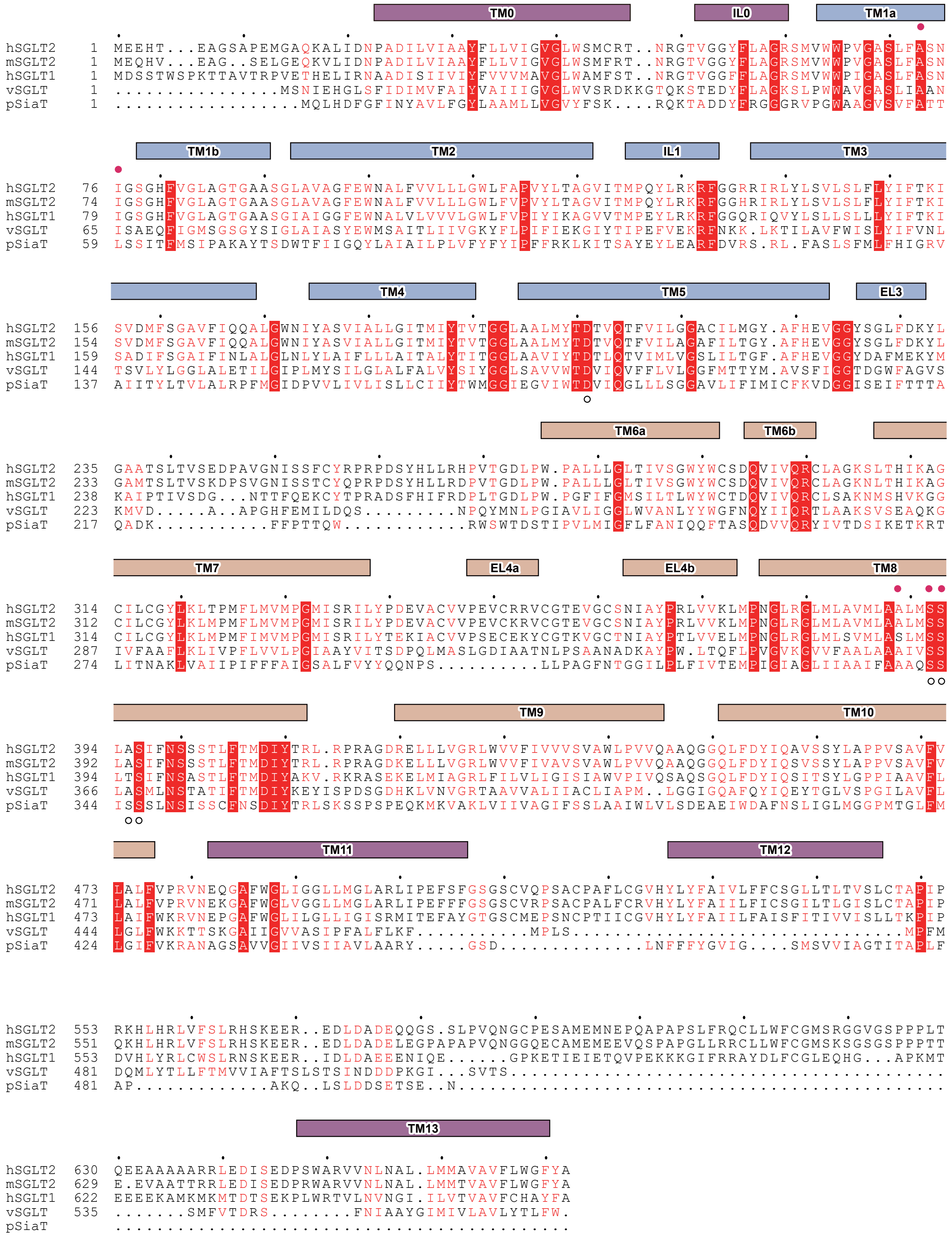

Supplementary Fig.1 Sequence alignment of SGLT.

Sequence alignment of hSGLT2 (UniProt: P31639), mSGLT2 (UniProt: Q92317), hSGLT1 (UniProt: P13866), *Vibrio parahaemolyticus* SGLT (UniProt: P96169), and Proteus mirabilis HI4320 sialic acid symporter (UniProt: B4EZY7), performed using Clustal Omega. Conserved transmembrane helices of hSGLT2 are indicated above the sequences. Similarly conserved residues are indicated by red letters. The residues at the conserved Na2 and Na3 sites of SGLT are highlighted with pink circles above or white circles below the alignments, respectively.

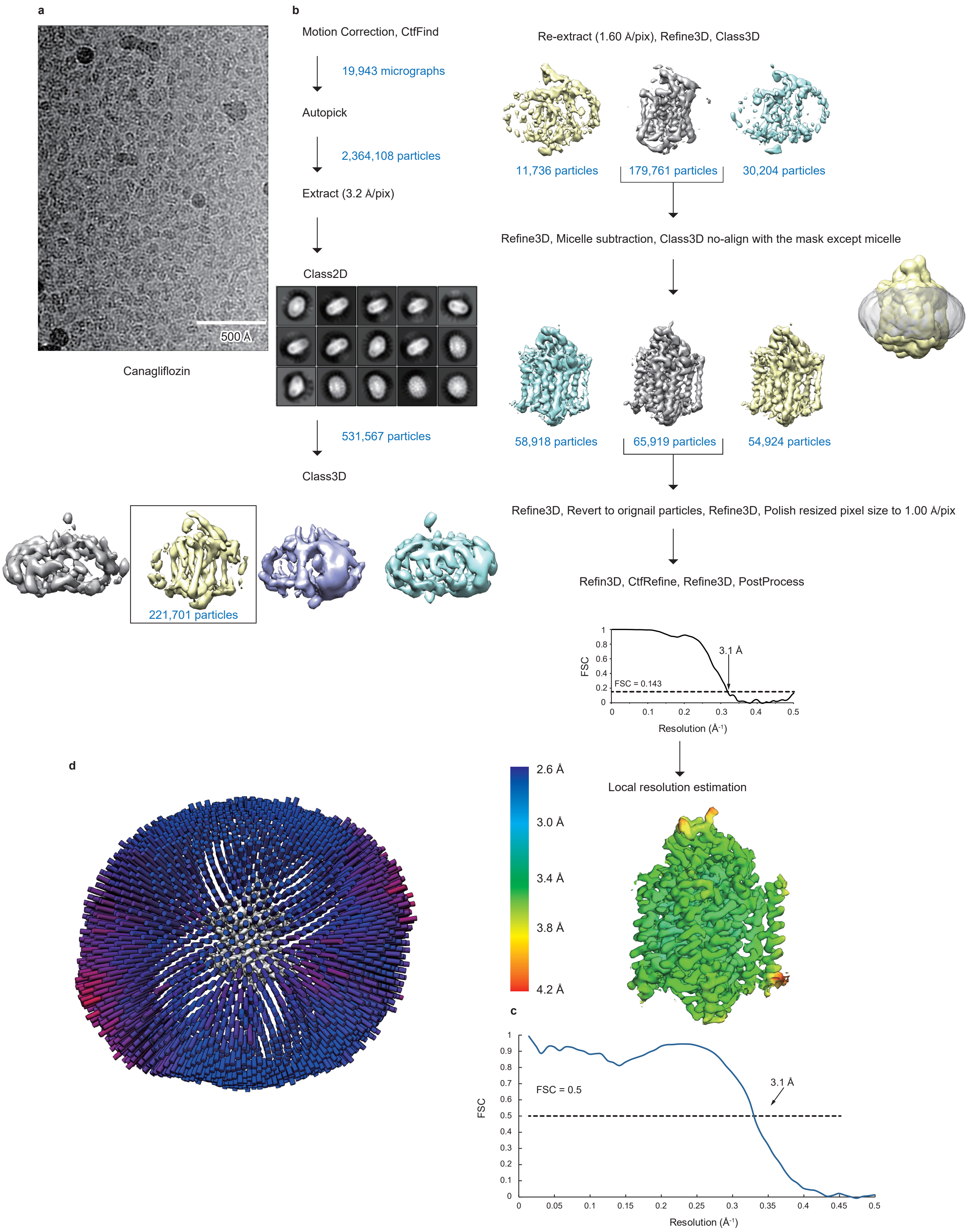

Supplementary Fig.2 Data processing of the canagliflozin-bound state.

a, Representative cryo-EM image of the hSGLT2–MAP17 complex in the presence of canagliflozin. b, Data processing workflow of single-particle image-processing and local-resolution analysis. Particles were separated into three groups via non-aligned 3D classification, with the mask (without micelles) shown in yellow. c, Cross-validation FSC curves for map-to-model fitting. d, Angular distributions of the final reconstruction.

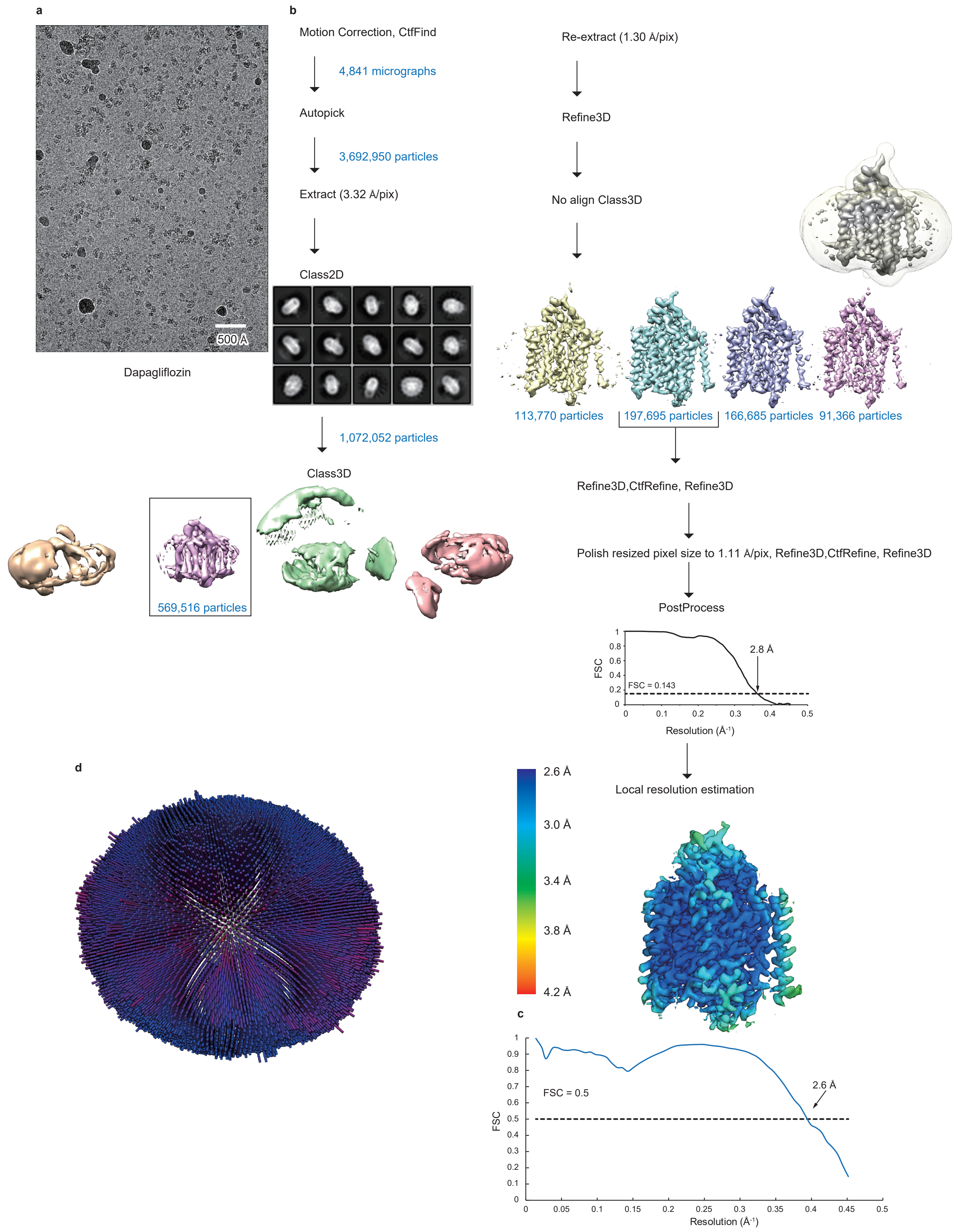

Supplementary Fig.3 Data processing of the dapagliflozin-bound state.

a, Representative cryo-EM image of the hSGLT2–MAP17 complex in the presence of dapagliflozin. b, Data processing workflow of single-particle image-processing and local-resolution analysis. Particles were separated into four groups via non-aligned 3D classification, with the mask (without micelles) shown in transparent white. c, Cross-validation FSC curves for map-to-model fitting. d, Angular distributions of the final reconstruction.

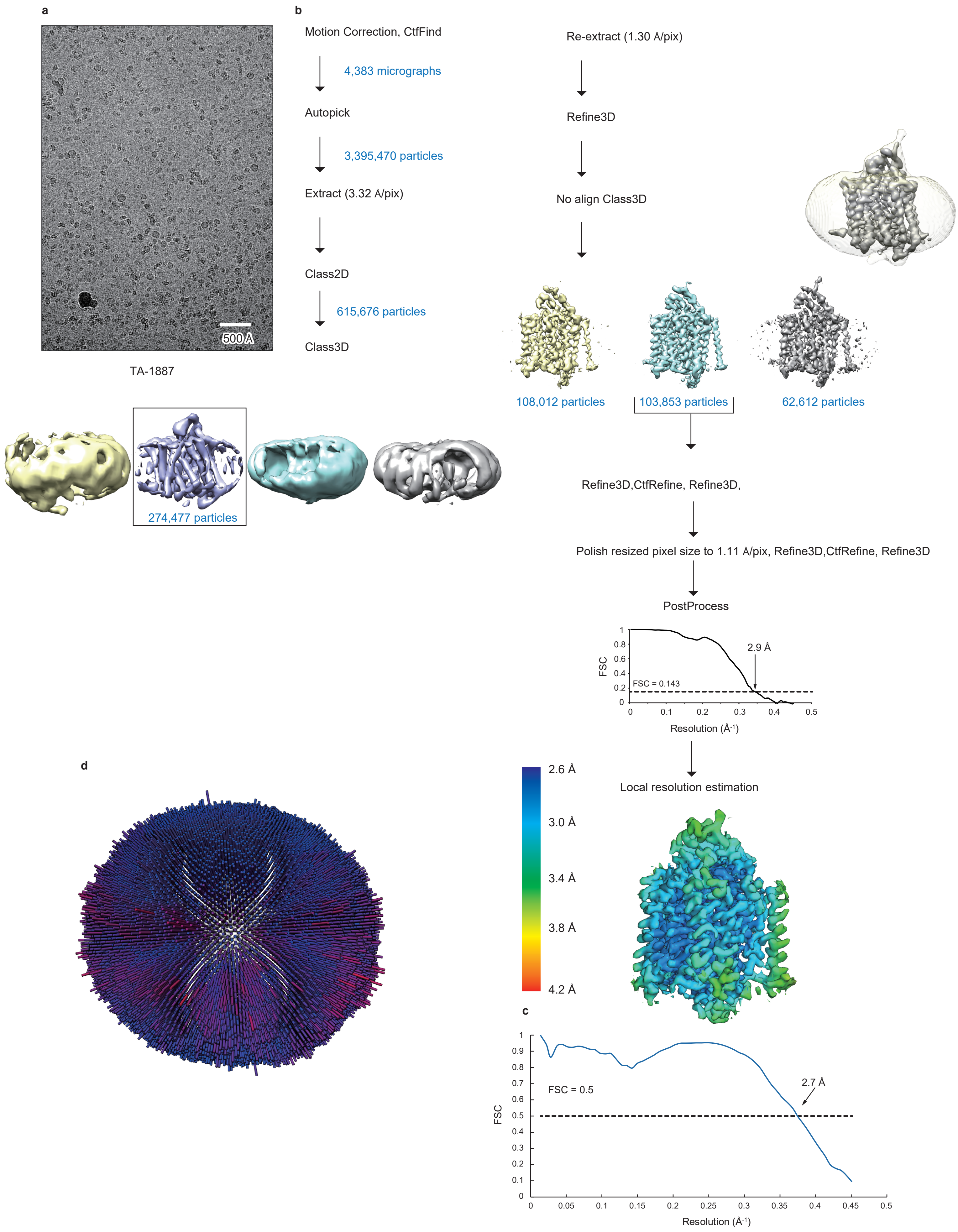

Supplementary Fig.4 Data processing of the TA-1887-bound state.

a, Representative cryo-EM image of the hSGLT2–MAP17 complex in the presence of TA-1887. b, Data processing workflow of single-particle image-processing and local-resolution analysis. Particles were separated into three groups via non-aligned 3D classification, with the mask covering the proteins and micelle shown in transparent white. c, Cross-validation FSC curves for map-to-model fitting. d, Angular distributions of the final reconstruction.

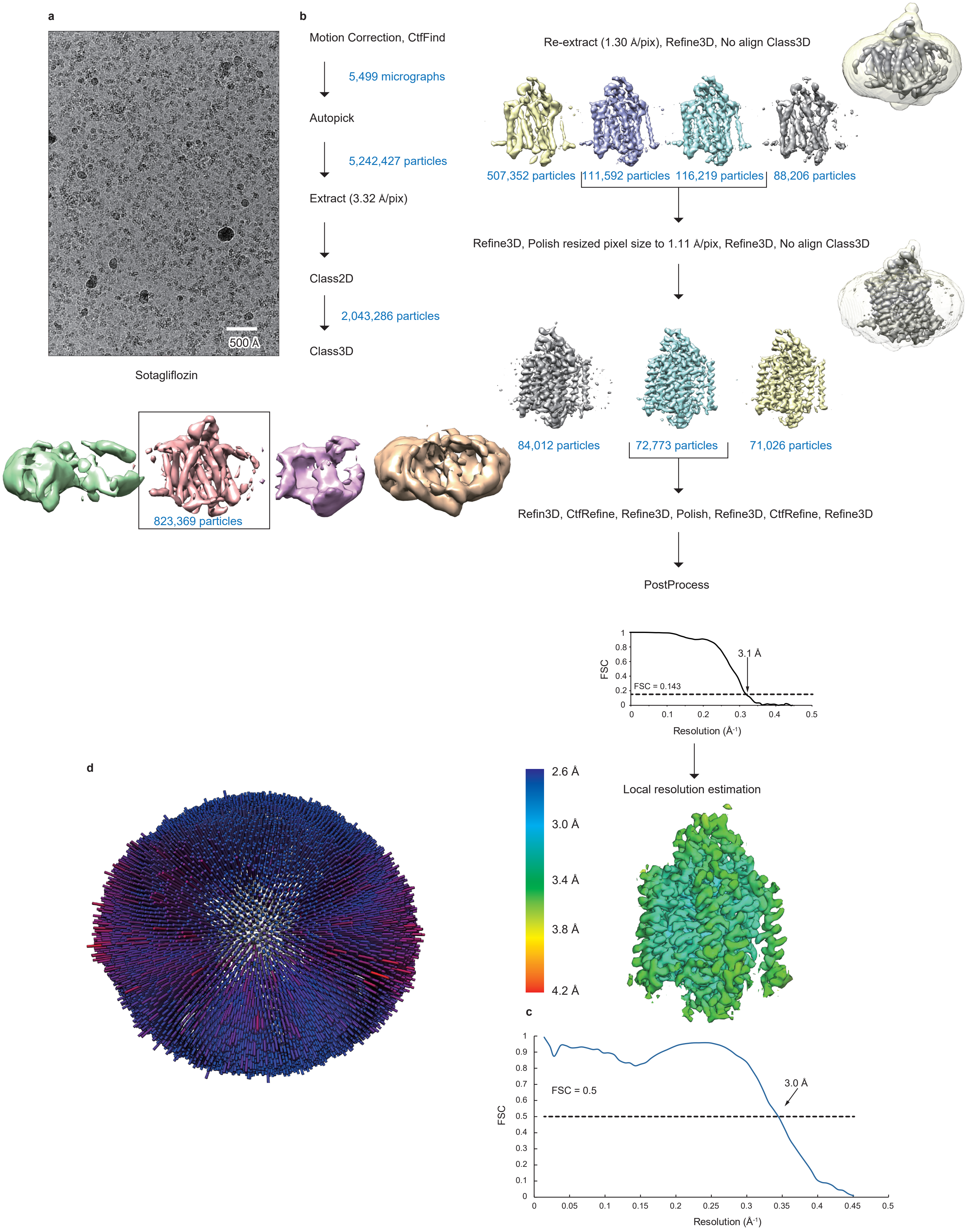

Supplementary Fig.5 Data processing of the sotagliflozin-bound state.

a, Representative cryo-EM image of the hSGLT2–MAP17 complex in the presence of sotagliflozin. b, Data processing workflow of single-particle image-processing and local-resolution analysis. Particles were separated into three groups via two rounds of non-aligned 3D classification, with the mask covering the proteins and micelles shown in transparent white. c, Cross-validation FSC curves for map-to-model fitting. d, Angular distributions of the final reconstruction.

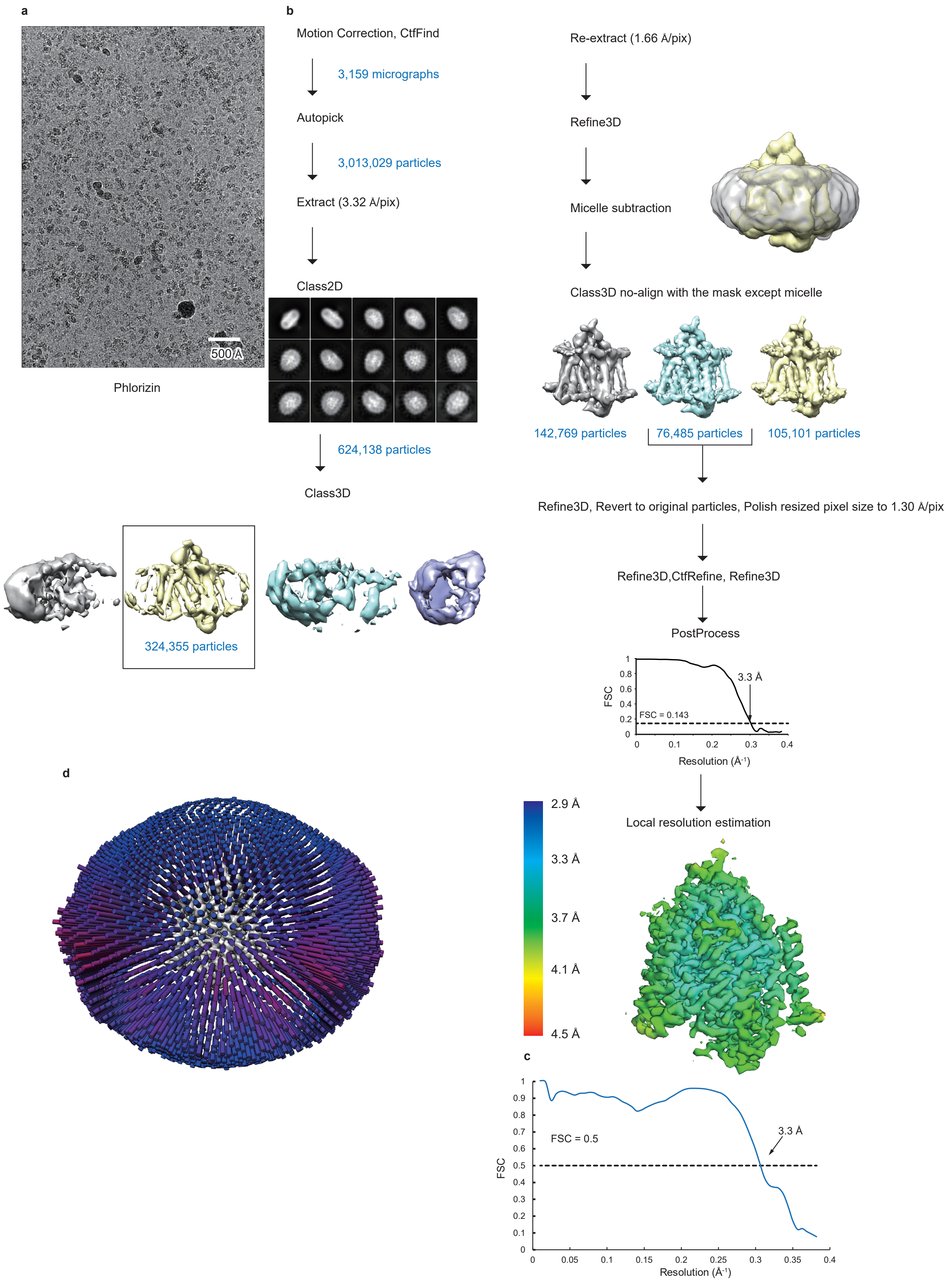

Supplementary Fig.6 Data processing of the phlorizin-bound state.

a, Representative cryo-EM image of the hSGLT2–MAP17 complex in the presence of phlorizin. b, Data processing workflow of single-particle image-processing and local-resolution analysis. Particles were separated into three groups via non-aligned 3D classification, with the mask covering the proteins (without micelles) shown in yellow. c, Cross-validation FSC curves for map-to-model fitting. d, Angular distributions of the final reconstruction.

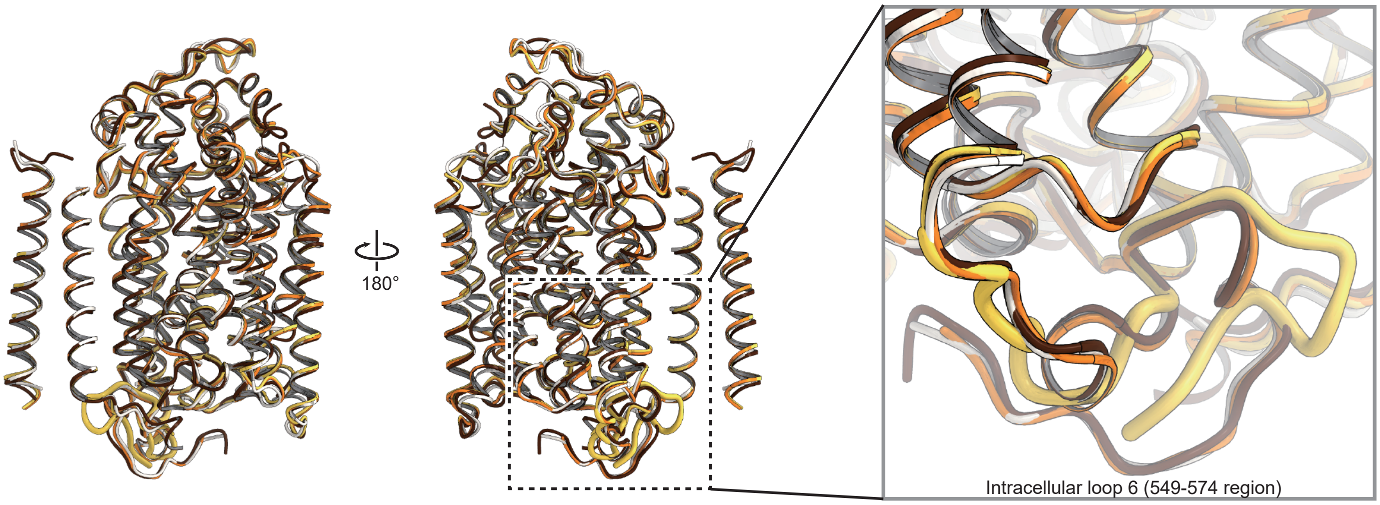

Supplementary Fig.7 Outward-facing SGLT1/2 superposition.

Brown: SGLT1-LX2761 (PDB ID: 7WMV), Grey: SGLT2-empagliflozin (PDB ID: 7VSI), Yellow: SGLT2-dapagliflozin (this study; 8HEZ), Orange: SGLT2-canagloflozin (this study; 8HDH). All structures are superimposed well [RMSD (Å) = 1.09-1.23].

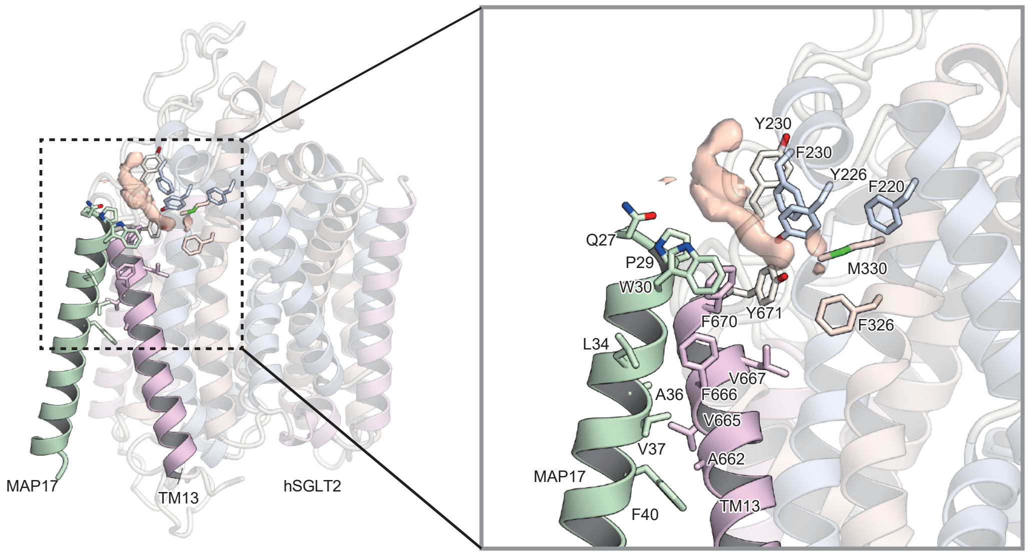

Supplementary Fig.8 MAP17 and SGLT2 interaction site.

The density of the lipid molecule (orange) is observed between MAP17 and SGLT2.

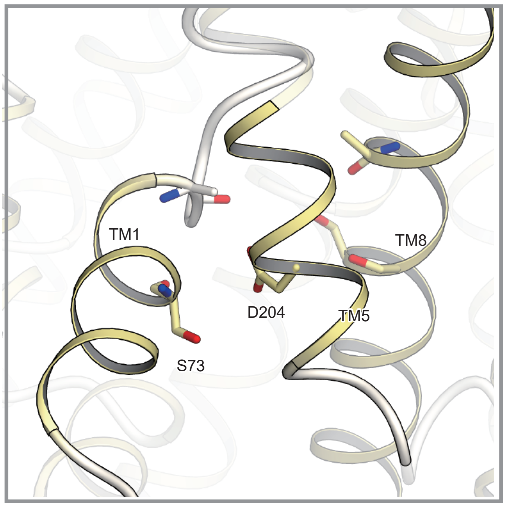

hSGLT1 (PDB : 7sla)

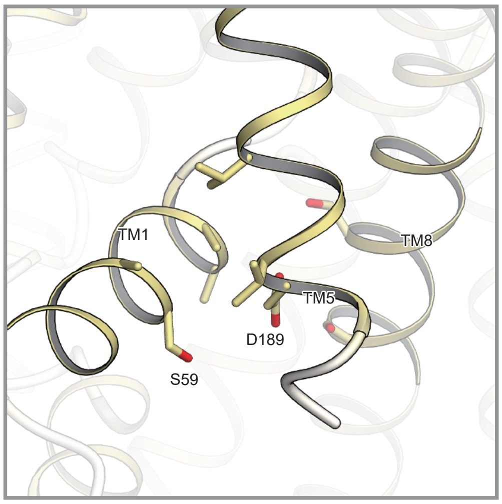

vSGLT (PDB : 3dh4)

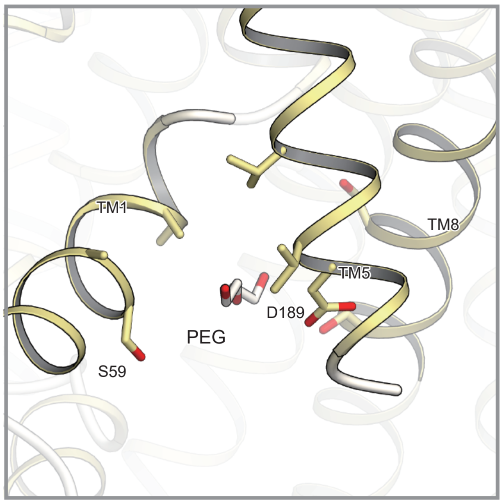

vSGLT (PDB : 2xq2)

Supplementary Fig.9 Comparison of the inward conformation of other SGLT structures.

Structural comparison of sites corresponding to the intracellular phlorizin-binding sites of SGLT2. From left to right, inward-occluded conformation of hSGLT1, inward-occluded conformation of vSGLT, and inward-open conformation of vSGLT. The structures are viewed from the membrane side.

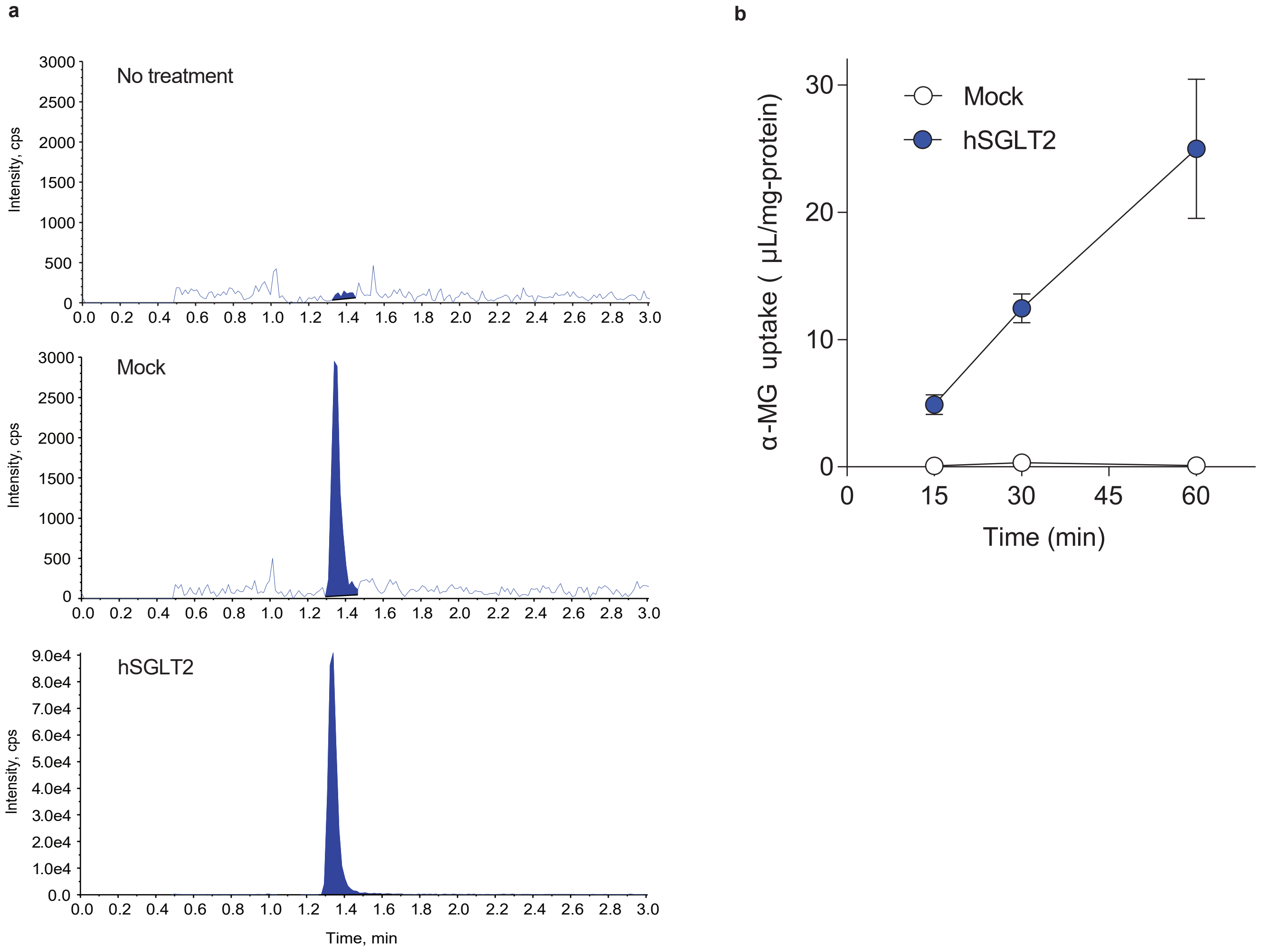

Supplementary Fig.10 Profiling of α-MG transport in hSGLT2-MAP17 expressing cells.

a, Chromatograms of α-MG in the lysates of untreated mock cells (No treatment), mock cells incubated with α-MG (Mock), and hSGLT2- and MAP17-expressing cells incubated with α-MG (hSGLT2). b, Time-course of hSGLT2-mediated α-MG uptake. α-MG uptake (500 μM) by hSGLT2-expressing cells and mock cells was examined. Each point represents mean ± SEM (n = 3, biological replicates).

Supplementary Table 1. Plasmids used in this study

Plasmid name	Expressed protein	Number	Promoter	Vector	Source
pcDNA3.4_N-terminal-EGFP_humanSGLT2	EGFP-hSGLT2	HM112	CMV	pcDNA3.4	This study
pcDNA3.4_C-terminal-EGFP_humanSGLT2	hSGLT2-EGFP	HM117	CMV	pcDNA3.4	This study
pcDNA3.4_humanMAP17	hMAP17	HM457	CMV	pcDNA3.4	This study
pcDNA3.4_N-sifnalPS_sfGFP_HRV3C_humanSGLT2	sfGFP-hSGLT2	HM464	CMV	pcDNA3.4	This study
pcDNA3.4_N-sifnalPS_sfGFP_HRV3C_humanSGLT2_S74A	sfGFP-hSGLT2_S74A	HM466	CMV	pcDNA3.4	This study
pcDNA3.4_N-sifnalPS_sfGFP_HRV3C_humanSGLT2_D201A	sfGFP-hSGLT2_D201A	HM467	CMV	pcDNA3.4	This study
pcDNA3.4_N-sifnalPS_sfGFP_HRV3C_humanSGLT2_F98A	sfGFP-hSGLT2_F98A	HM471	CMV	pcDNA3.4	This study
pcDNA3.4_N-sifnalPS_sfGFP_HRV3C_humanSGLT2_F453A	sfGFP-hSGLT2_F453A	HM472	CMV	pcDNA3.4	This study

Suplementary Table 2. Primers used in this study

No	Primer name	Sequence 5'->3'
1	C-terminal-EGFP_pcDNA3.4_vector_amplify_R	AAGGGTTCGATCCTCTAGAGTCCGGAG
2	C-terminal-EGFP_pcDNA3.4_vector_amplify_F	CTCGAGGAAAACCTGTATTTTCAGGGCGGC
3	N-terminal-EGFP_pcDNA3.4_vector_amplify_R	GAATTCTCGAGAGCCCTGAAAGTACAGGTTTTCTC
4	N-terminal-EGFP_pcDNA3.4_vector_amplify_F	TAATGAAGGGTTCGATCCCTACCGGTTAGTAATG
5	C-terminal-EGFP-Human SGLT2_insert_F	GAGGATCGAACCCTTGCCGCCACCATGGAGGAACATACCGAAGC
6	C-terminal-EGFP-Human SGLT2_insert_R	CAGGTTTTCCTCGAGAGCATAGAATCCCCAAAGAAAGAC
7	N-terminal-EGFP-Human SGLT2_insert_F	GGCTCTCGAGAATTCATGGAGGAACATACCGAAGC
8	N-terminal-EGFP-Human SGLT2_insert_R	TCGAACCCTTCATTAAGCATAGAATCCCCAAAGAAAGAC
9	human_MAP17_R	CGAACCCTTTCATTACATCGGGGTACTCCTCACTTTG
10	human_MAP17_F	GAGGATCGAACCCTTGCCGCCACCATGTCTGCCTTGAGCCTGC
11	insert_HRV3C _F	TTCCAGGGACCCGGCTCCATGGAGGAACATACCGAAGCTG
12	insert_HRV3C _R	CAGCACCTCGAGCCCGCTGAGTCCGGACTTGTACAGCTC
13	sfGFP_pcDNA3.4_vector_amplify_F	TAATGAAAGGGTTCGATCCCTACC
14	sfGFP_pcDNA3.4_vector_amplify_R	GAATTCGCCATGGGTAATTCCG
15	HRV3C_-Human SGLT2_insert_F	ACCCATGGCGAATTCAGCGGGCTCGAGGTGCTG
16	HRV3C_-Human SGLT2_insert_R	CGAACCCTTTCATTAAGCATAGAATCCCCAAAGAAAGAC
17	Human SGLT2_S74A_mutation_F	TTTGCCGCTAACATCGGAAGCGGCCAC
18	Human SGLT2_S74A_mutation_R	GATGTTAGCGGCAAACAGAGAGGCGCC
19	Human SGLT2_D201A_mutation_F	ATACAGTATACAGCCACTGTCCAGACCTTCGTGAT
20	Human SGLT2_D201A_mutation_R	AAGGTCTGGACAGTGGCTGTATACATCAGGGCTGC
21	Human SGLT2_F98A_mutation_F	GTCGCAGGGGCTGAGTGGAACGCTCTGTTTG
22	Human SGLT2_F98A_mutation_R	GTTCCACTCAGCCCCTGCGACAGCCAGCCC
23	Human SGLT2_F453A_mutation_F	GGACAACTCGCCGACTACATCCAGGCAGTG
24	Human SGLT2_F453A_mutation_R	GATGTAGTCGGCGAGTTGTCCGCCCTGGGC
