# Supplementary material for: Transport and inhibition mechanism of the human SGLT2–MAP17 glucose transporter
Source: Nat Struct Mol Biol. 2023 Dec 6;31(1):159–69. doi: 10.1038/s41594-023-01134-0 (PMC10803289; doi:10.1038/s41594-023-01134-0)
Supplement: Supplementary file 2 — Reporting Summary [file 41594_2023_1134_MOESM2_ESM.pdf]

Corresponding author(s): Masahiro Hiraizumi
Osamu Nureki
Ikuko Miyaguchi

Last updated by author(s): Jul 21, 2023

Reporting Summary

Nature Portfolio wishes to improve the reproducibility of the work that we publish. This form provides structure for consistency and transparency in reporting. For further information on Nature Portfolio policies, see our Editorial Policies and the Editorial Policy Checklist.

Statistics

For all statistical analyses, confirm that the following items are present in the figure legend, table legend, main text, or Methods section.

n/a	Confirmed	
☐	☒	The exact sample size (*n*) for each experimental group/condition, given as a discrete number and unit of measurement
☐	☒	A statement on whether measurements were taken from distinct samples or whether the same sample was measured repeatedly
☒	☐	The statistical test(s) used AND whether they are one- or two-sided<br>*Only common tests should be described solely by name; describe more complex techniques in the Methods section.*
☒	☐	A description of all covariates tested
☒	☐	A description of any assumptions or corrections, such as tests of normality and adjustment for multiple comparisons
☐	☒	A full description of the statistical parameters including central tendency (e.g. means) or other basic estimates (e.g. regression coefficient) AND variation (e.g. standard deviation) or associated estimates of uncertainty (e.g. confidence intervals)
☒	☐	For null hypothesis testing, the test statistic (e.g. *F*, *t*, *r*) with confidence intervals, effect sizes, degrees of freedom and *P* value noted<br>*Give P values as exact values whenever suitable.*
☒	☐	For Bayesian analysis, information on the choice of priors and Markov chain Monte Carlo settings
☒	☐	For hierarchical and complex designs, identification of the appropriate level for tests and full reporting of outcomes
☒	☐	Estimates of effect sizes (e.g. Cohen's *d*, Pearson's *r*), indicating how they were calculated

Our web collection on statistics for biologists contains articles on many of the points above.

Software and code

Policy information about availability of computer code

Data collection	EPU (version 1.19), Serial EM (version 3.7.10), Analyst 1.6.2.
Data analysis	RELION (version 3.1 and version 4.0), Servalcat (version 0.2.0), MOLREP (version 11.7), COOT (version 0.8.9.1), UCSF Chimera (version 1.14), CueMol2 (http://www.cuemol.org/ version 2.2.3.443), GraphPad Prism 8.4.3., REFMAC5(version 5.8.0267)

For manuscripts utilizing custom algorithms or software that are central to the research but not yet described in published literature, software must be made available to editors and reviewers. We strongly encourage code deposition in a community repository (e.g. GitHub). See the Nature Portfolio guidelines for submitting code & software for further information.

Data

Policy information about availability of data

All manuscripts must include a data availability statement. This statement should provide the following information, where applicable:

- Accession codes, unique identifiers, or web links for publicly available datasets
- A description of any restrictions on data availability
- For clinical datasets or third party data, please ensure that the statement adheres to our policy

Cryo-EM density maps were deposited in the Electron Microscopy Data Bank under accession codes EMD-34673 (canagliflozin-bound state), EMD-34705 (dapagliflozin-bound state), EMD-34610 (TA-1887-bound state), EMD-34737 (sotagliflozin-bound state), and EMD-34823 (phlorizin-bound state). Atomic

coordinates have been deposited in the Protein Data Bank under IDs 8HDH (canagliflozin-bound state), 8HEZ (dapagliflozin-bound state), 8HB0 (TA-1887-bound state), 8HG7 (sotagliflozin-bound state), and 8HIN (phlorizin-bound state). Source data are provided with the paper.

Research involving human participants, their data, or biological material

Policy information about studies with human participants or human data. See also policy information about sex, gender (identity/presentation), and sexual orientation and race, ethnicity and racism.

Reporting on sex and gender	not relevant
Reporting on race, ethnicity, or other socially relevant groupings	not relevant
Population characteristics	not relevant
Recruitment	not relevant
Ethics oversight	not relevant

Note that full information on the approval of the study protocol must also be provided in the manuscript.

Field-specific reporting

Please select the one below that is the best fit for your research. If you are not sure, read the appropriate sections before making your selection.

☒ Life sciences ☐ Behavioural & social sciences ☐ Ecological, evolutionary & environmental sciences

For a reference copy of the document with all sections, see nature.com/documents/nr-reporting-summary-flat.pdf

Life sciences study design

All studies must disclose on these points even when the disclosure is negative.

Sample size	No statistical method was used to determine the sample size. For cryo-EM analyses, sample sizes were determined by the availability of microscope time and the number of particles on electron microscopy grids enough to obtain a structure at the reported resolution. For transporter functional analyses, no sample size determination analysis was determined as binding assay and uptake assay got consistent signal and low background to ensure the reproducibility and no statistical analysis was performed. These assays are performed with similar sample sizes in previous studies as well.
Data exclusions	For cryo-EM analyses, particles that did not contribute to improving map quality were excluded following the standard classification procedures in RELION. This is standard practice for structure determination by cryo-EM.
Replication	For cryo-EM analyses, related experiments including FSEC, purification, and SDS-PAGE were reproduced at least two times and structure determination was completed once. In transporter functional analyses, three or four technical replicates of binding assay and three or four biologically independent replicates of uptake assay was evaluated and all attempts at replication were successful.
Randomization	For cryo-EM analyses, particles were randomly assigned to half-maps for resolution determination following the standard procedures in RELION. For transporter functional analyses, randomization was not performed since samples were not divided into two or more groups.
Blinding	For cryo-EM analyses and transporter functional analyses, blinding was not applicable since this type of studies does not use group allocation.

Reporting for specific materials, systems and methods

We require information from authors about some types of materials, experimental systems and methods used in many studies. Here, indicate whether each material, system or method listed is relevant to your study. If you are not sure if a list item applies to your research, read the appropriate section before selecting a response.

Materials & experimental systems

n/a	Involved in the study
☒	☐ Antibodies
☐	☒ Eukaryotic cell lines
☒	☐ Palaeontology and archaeology
☒	☐ Animals and other organisms
☒	☐ Clinical data
☒	☐ Dual use research of concern
☒	☐ Plants

Methods

n/a	Involved in the study
☒	☐ ChIP-seq
☒	☐ Flow cytometry
☒	☐ MRI-based neuroimaging

Eukaryotic cell lines

Policy information about cell lines and Sex and Gender in Research

Cell line source(s)	HEK293 (ECACC) and Expi293 (Thermo Fisher Scientific)
Authentication	No further authentication was performed after receipt.
Mycoplasma contamination	Not performed
Commonly misidentified lines (See ICLAC register)	No commonly misidenfied cell lines were used.
