# Supplementary figures and images for: Transport and inhibition mechanism of the human SGLT2–MAP17 glucose transporter
Source: Nat Struct Mol Biol. 2023 Dec 6;31(1):159–69. doi: 10.1038/s41594-023-01134-0 (PMC10803289; doi:10.1038/s41594-023-01134-0)

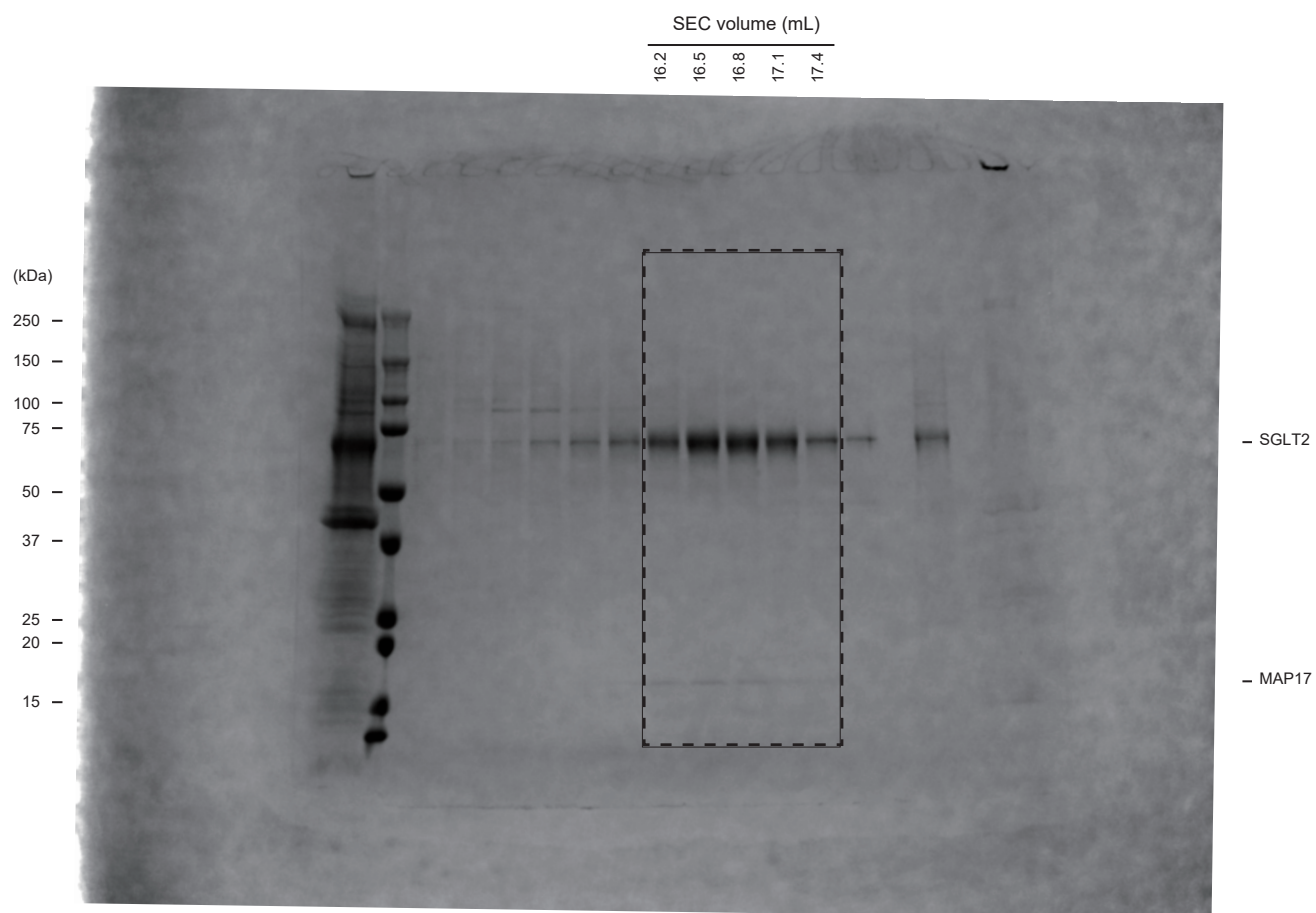

Uncropped gels

Supplement: Supplementary file 10 — Uncropped gel images for Extended Data Fig. 1c. [file 41594_2023_1134_MOESM10_ESM.pdf]
